# A scoping review of physical activity and screen time guidelines for use in Outside School Hours Care

**DOI:** 10.1186/s12887-020-02352-x

**Published:** 2020-10-06

**Authors:** Rosa Virgara, Lucy Lewis, Anna Phillips, Mandy Richardson, Carol Maher

**Affiliations:** 1 Allied Health and Human Performance Unit, University of South Australia, Adelaide, Australia; 2 Caring Futures Institute, College of Nursing and Health Sciences, Flinders University, Bedford Park, Australia; 3 OSHC SA Chairperson, NOSHA SA Branch, Adelaide, Australia

**Keywords:** Guidelines, Outside school hours care, Physical activity, Screen time, After school

## Abstract

**Background:**

Globally, millions of children attend Outside School Hours Care. Children’s activity in this setting is critical to meeting daily physical activity recommendations. Guidelines are evidence-based statements intended to optimise practice. This study aimed to identify guidelines used in Outside School Hours Care for physical activity and screen time and summarise their content and methods of development.

**Methods:**

Outside School Hours Care guidelines for children aged 5 to 12 years were identified by systematically searching Medline, Emcare, Embase, Scopus, ERIC, Sportsdiscus, TROVE, ProQuest, UpToDate, NICE, SIGN and Google in accordance with PRISMA-ScR guidelines. The search was conducted in March 2019 and results screened independently by two authors. Data were synthesized narratively.

**Results:**

Nine guideline documents were identified from grey literature only (*n* = 8 USA, *n* = 1 Canada). The included guidelines focused predominantly on the after school care period (*n* = 9 vs *n* = 2 for the before school period). All had recommendations for physical activity, whilst 7 included screen time recommendations. There was considerable variability across the physical activity and screen time recommendations, though taken together, they recommended 30–60 min of moderate- to- vigorous physical activity and less than 60 min of recreational screen time per session. All guidelines were developed by expert/stakeholder panels, but none followed rigorous guideline development methods.

**Conclusions:**

Limited published guidelines for physical activity and screen time in Outside School Hours Care exist. There is a variation in duration and intensity of physical activity recommended, whilst screen time recommendations are more consistent. Guidelines designed with rigorous tools and for other world regions are warranted.

## Background

### Rationale

Physical activity (PA) is associated with an array of health benefits across the lifespan, such as improved cardiovascular health, reduced rates of obesity, cancer and other non-communicable diseases [1]. In children aged 5–17 years, specifically, it has been linked with improved body composition, cardiovascular and metabolic health, cardiorespiratory and musculoskeletal fitness, academic achievement and cognition, improved mental health and quality of life, emotional regulation and social behaviour [2]. In addition, excessive recreational screen time has been associated with a wide range of physical and mental health deficits [3, 4]. Since around the 2000s, PA and sedentary behaviour (including recreational screen time) have been viewed as being “independent” health behaviours, with independent health impacts. However, more recently, the field has recognised that daily activities are constrained within the 24-h day, and that more time on one activity must necessarily mean less time for something else, leading to a “whole-of-day” or integrated approach [5]. In keeping with this paradigm shift, the latest iterations of children’s PA guidelines published by the World Health Organization, and various countries including Canada [6], Australia [7], New Zealand [8], South Africa [9], Finland [10] and Croatia [11] recommend that each day children aged 5–17 years should get at least 60 min of moderate- to- vigorous physical activity (MVPA), no more than 2 h of recreational screen time and between 9 and 11 h of sleep for children aged 5–13 years, or 8–10 h of sleep for those aged 14–17 years [12].

Despite these clear and achievable guidelines, the most recent PA global matrix conducted in 2018 identified that that only 40 to 46% of children between the ages of 5 and 17 years in high income countries (such as the United States, Australia, New Zealand, United Kingdom and Spain) were achieving the recommended levels of PA [13]. Low- to middle- income countries and high income countries had similar low PA prevalence of 40–46% and 34–39% respectively [14]. Results are similar for screen time use. The same global matrix identified that in children from high and very high income countries only 27–39% were adhering to the guideline of no more than 2 h recreational screen time use [14]. This was consistent with earlier findings from Atkin in 2014 [15] which found approximately two thirds of children exceeded the screen time guidelines. Given this low prevalence of sufficient PA, and high prevalence of excessive recreational screen time, further efforts are required to identify ways to help children achieve healthy daily MVPA and screen time.

Many of these efforts have been during the school period as way of reaching children in an equitable way [16]. However the school day only contributes less than half the total daily target for children aged 5–17 years; and with increasing academic pressures there is less time available for school based PA interventions [17–19]. Another period of the day which has the ability to make a substantial contribution to children’s activity patterns is the outside school hours period [18, 20]. This refers to the time before and after school hours, on weekends and school holidays. It has been identified as a potential period to improve PA and combat childhood obesity [19]. A study of Australian children (mean age 8.1 years) found that the after school period (between the hours of 3 – 6 pm) accounted for 30% of children’s total daily MVPA, 25% of their daily light PA and 80% of their total daily recreational screen time [21].

Many primary or elementary school aged children (5–12 years of age) attend formal childcare before and after school, and during school holidays in services referred to as Outside School Hours Care (OSHC) [22]. This is partly due to changing societal trends, including an increasing number of families with two working parents, single parent families, and reduced childcare support from extended families [23]. Recent estimates suggest that, in the United States, 18% of school aged children attend after school programs [24]. In Australia, nearly 10% of primary school aged children (5–12 years) attend before and/or after school childcare services [25]. Given the growing numbers of children who attend these services, providing specific MVPA and screen time recommendations for use in OSHC, may improve practice and positively influence activity behaviours to help children achieve the 24-h guideline recommendations [26].

To our knowledge, no previous studies have attempted to identify guidelines addressing PA and/or screen time in the OSHC setting. Beets et al. [19] conducted a related review which attempted to identify documentation relating to PA promotion for US-based after school program providers. By identifying current standards and policy, Beets and colleagues hoped the review would allow “the compilation of baseline standards and policies that could be tested empirically, with the results of such investigations used to develop national guidelines” p.412 [19]. Their review found 47 states had an after school program policy of which 14 US states had after school program documentation incorporating language about promoting PA. Five of those 14 states specified actual durations of time that children should be active. Beets reported that these were only published in grey literature sources [19].

Given the importance of children’s PA and screen time behaviours in the before and after school periods can have on achieving the 24-h movement guidelines; coupled with the role that guideline documents can play in promoting healthy practice, we set out to review the current state of the international literature regarding guidelines for PA and screen time in the OSHC setting.

### Objectives

This scoping review aimed to determine the published guidelines that exist for PA and/or screen time for OSHC and the methods used to create the guidelines. Specifically, we aimed to answer the following research questions:

1. What published guidelines currently exist for PA and/or screen time specifically for use in OSHC?
  - Are they still in use?
  - Are they endorsed or implemented by Government authorities?
2. What do these guidelines recommend?
  - How much PA do they encourage?
  - How much screen time do they permit?
3. What methodological processes were followed to create these guidelines?

The aim of this scoping review was not to assess these guidelines for methodological rigour, but rather understand the content and processes used to create such guidelines.

## Methods

### Protocol and registration

The protocol for this review was prospectively registered (JBI database, registered 26.3.19 at https://joannabriggs.org/research/registered_titles.aspx). The scoping review was undertaken in accordance with the Preferred Reporting Items for Systematic Reviews and Meta-analysis (PRISMA-ScR) guidelines for scoping reviews [27]. Ethics approval was not required for this review.

### Eligibility criteria

To be eligible, the guidelines needed to refer to PA and/or screen time behaviours of primary/elementary school aged children (aged between approximately 5 and 12 years) specifically in OSHC setting. This age range was selected as children aged 13 and over are commonly in high school and do not access OSHC.

The guidelines had to include specific recommendations for PA and/or screen time, in the OSHC setting. The guidelines must have been for use in the OSHC setting (in the hours of the day before and after formal school lessons or school holidays), in a formal childcare setting (as opposed to informal childcare provided by a family member), and published by an authoritative organisation (e.g., Young Men’s Christian Association (YMCA), Government department etc.)

For the purpose of this review, to be considered a guideline, the document was required to have provided specific directives for the volume of PA, in terms of duration, with or without intensity (e.g., that children should achieve at least 30 min of MVPA) during OSHC. Statements that were worded generally (e.g., that children should be given opportunity for active play) were not considered guidelines and therefore not included. It was not required that the guideline provided specific details of the methods used for creation, however this information was also collected where available (e.g., if processes such as GRADE were followed or other guideline development tools).

In accordance with scoping review recommendations [28], *any* existing literature was considered for inclusion. This included, but was not limited to, quantitative journal articles/pieces of original research, theses, government (either state or national) reports/guidelines and non-government organisation or private sector guidelines published in grey literature also.

There were no exclusion criteria however database searches were only in English. All guidelines, relevant articles or studies, even if no longer currently in use, were considered for the review.

### Information sources

An initial scoping search was undertaken in March 2019 of six databases (MEDLINE, Emcare, Embase, Scopus, ERIC and Sportsdiscus). After this initial search, the key words and subject heading words from the sources identified as appropriate were added to the search strategy and searched across databases (Table 1). This search strategy was created in collaboration with an academic librarian. Reference lists of all included sources were screened for further potentially eligible guidelines.

**Table 1:** Search strategy for Medline

1. (“out of school care” or “OSHC” or “outside school hours care” or “after school” or “after-school” or after school or “before school” or “before-school” or before school) 2. Practice guideline^a^ 3. Guideline^a^ 4. (guideline^c^ or recommend^b^ or polic^b^) 5. 2 or 3 or 4 6. Exercise^a^ 7. exercis^b^.mp 8. physical activit^b^ 9. 6 or 7 or 8 10. Sedentary Behaviour^a^ 11. (sedentary or screen-time or screen time) 12. 10 or 11 13. 1 and 5 and 9 and 12	

Footnote: (^a^) Subject heading (MeSH) Medline, (^b^) truncation symbol, (^c^) wildcard symbol guideline AND physical activity OR screen time AND after school AND before school

The search strategy was adapted for use in grey literature (Table 2). The following sources were searched: Google, TROVE, ProQuest Dissertations and Theses, UpToDate, National Institute for Health and Clinical Excellence (NICE) and Scottish Intercollegiate Guidelines Network (SIGN). Grey literature was searched for the first 500 articles. Searches were stopped after 10 irrelevant articles were sequentially identified through the screening process. No geographical limits were placed on Google searches to identify as many international guidelines as possible.

**Table 2:** Search strategy for Grey literature e.g. Google

guideline AND physical activity OR screen time AND after school AND before school	

### Selection of sources of evidence

Results from the database and grey literature searches were collated and exported into Covidence software [29] to allow for removal of duplicates and screening.

Two authors (RV and LL) independently screened all results based on title and abstract in Covidence. Disagreements were discussed and resolved without requiring a third author. A flowchart in accordance with PRISMA-ScR [27] was created with reasons for exclusion recorded.

### Data charting

An Excel spreadsheet, as recommended by the Joanna Briggs Institute (JBI) [28], was used to table the data extraction from the included documents, including bibliographic details, document type, source, country of origin, sponsors, aims of guidelines, methods of development and guideline recommendations (Supplementary File 1). Data extraction was completed by the primary author (RV), with any discrepancies/uncertainties referred to a secondary author (LL). Information regarding who created the guidelines (e.g. government agencies, academics, researchers), how much PA and/or screen time is being advocated, what methods were followed to develop the guidelines, and whether/how the guidelines have been disseminated/implemented was also collected.

### Synthesis of results

Due to the descriptive nature of the extracted data, data were synthesised narratively by the primary author (RV) and cross checked by the authorship team.

## Results

### Selection of sources of evidence

**Fig. 1:**
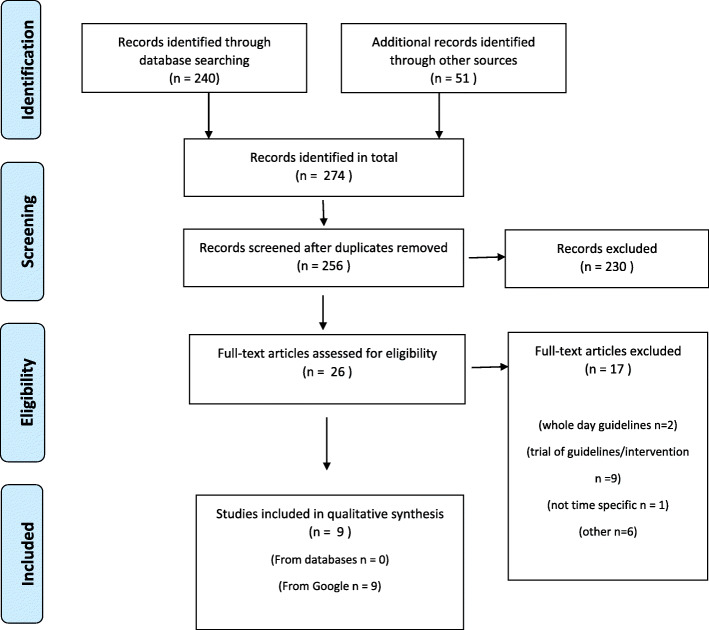
PRISMA-ScR Flow chart

A total of 274 citations were identified from the database and grey literature searches. 18 duplicates were removed, and 256 title and abstract screening. Of those, 26 were reviewed in full text and nine were included in the final scoping review (Fig. 1). All nine included guidelines were identified through online grey literature sources; none of the guidelines were published in scientific, peer-reviewed sources. Seventeen documents were excluded due to a lack of time-specific guidelines for use in the OSHC setting e.g. only providing guidelines for the whole day rather than specific for OSHC setting or referring to ways to improve PA during the school day in lesson breaks such as recess and lunch.

### Characteristics of included documents

Of the nine documents included [30–38], eight originated from the United States and one from Canada. These documents all targeted the after school care period, with only 2 documents also targeting the before school period [30, 31]. Table 3 provides a detailed summary of the included guideline documents and Table 4 the specific PA and/or screen time recommendations from those guideline documents.

**Table 3:** Summary of included guidelines for PA and/or screen time in the after school period

Year	Reference	Guideline Title	Country of Origin	Source	Funding	Aim of document	Age Group
2018	National Afterschool Alliance	The National Afterschool Alliance HEPA Standards 2.0	U.S.	Government document available online	National Afterschool Association	“NAA adopted the HEPA standards to provide practical, comprehensive guidance for OST programs. The NAA HEPA Standards address food and beverage and physical activity content and quality, staff training, social supports (including staff role modelling, family engagement, and children’s social development), program support, and environmental support.” p. 3	Not specified
2018	Ontario Ministry of Education	Before-And-After School Programs (Kindergarten to Grade 6) - Policies and Guidelines for School Boards	Canada	Government document available online	Ministry for Education – Ontario, Canada	“This document summarizes the provisions set out in the Education act and regulations for before-and-after school programs for students in Kindergarten to Grade 6. It also sets out requirements with regard to reporting and program content for before-and-after school programs and additional considerations to support the implementation of these programs” p.1	Kindergarten – Grade 6
2014	Public Health Law Centre	Minnesota Afterschool and OST - Best Practice Guidelines	U.S.	Public health document available online	Public Health Law Centre at Williams Mitchell College of Law, Minnesota	“Guidelines developed by national and regional experts to advance best practices for healthy eating and physical activity as part of a comprehensive strategy to prevent childhood obesity. The guidelines provide practical standards to help afterschool/OST programs: 1) improve the nutrition of snacks and meals; and 2) select activities and play spaces that will increase physical fitness.” p. 3	Not specified
2011	Ohio Afterschool Network and Ohio Department of Health	Ohio Kids on the Move: Physical Activity Guidelines for Afterschool Programs	U.S.	Public health document available online	Ohio Department of Health and Ohio Afterschool Network	“These guidelines are not requirements, but are rather recommendations designed to support afterschool programs as they address the critical issue of childhood obesity. This document aims to identify and define the areas of physical activity in which staff and caregivers of children grades K^− 12^ can strive to gain” p.8	Not specified
2011	National Afterschool Alliance	National Afterschool Association HEPA Standards	U.S.	Government document	National Afterschool association	“In 2011 the National Afterschool Association adopted standards for HEPA in Out-of-School time” p.1	Not specified
2010	Harvard T.H. Chan School of Public Health	OSNAP	U.S.	University document from a prevention research centre available online	YMCA of USA	“The OSNAP goals for nutrition and physical activity aim to help program leaders create healthier out-of-school environments for children. They are based on current scientific evidence about healthy eating and physical activity and have been developed for out-of-school settings like sport programs and afterschool programs, and can easily be modified for full-day programs like summer camps.” p.1 In addition to this, it provides another resource on i5w website, called “Food and Fun program”, which outlines the same guidelines as set out by the OSNAP initiative.	In published articles related to guidelines states children 5–12 years
2009	Californian Department of Educaiton	California After School Physical Activity Guidelines	U. S	Government document available online	n/a	“… to make available to after school providers a resource for implementing physical activity in their after school programs” p. iv	Not specified
2009	Move More After School Collaboration	Move More North Carolina: Recommended standards for After School Physical Activity	U.S.	Government document available online	North Carolina Cap WakeMed Boys and Girls Clubs - North Carolina, Alliance North Carolina Recreation and Park Association, North Carolina Health and Wellness Trust Fund, North Carolina Public Health, East Carolina University, North Carolina Afterschool Coalition, North Carolina Department of Juvenile Justice and Delinquency Prevention, Public Schools of North Carolina, North Carolina PTA	“Move More North Carolina: Recommended Standards for After-School Physical Activity outlines recommendations for providing quality physical activity in North Carolina after-school programs. The standards are based on current research and expert opinion on how after-school programs can help young people move more. It will take many people working together to put the standards into practice in after-school programs across the state.” p. 2	States children and adolescents in introduction, but provides physical activity suggestions for elementary aged children
2008	New York State Department of Health	*Healthy Kids, Healthy New York - After-School initiative toolkit*	U.S.	Public health document available online	New York State Healthy Eating and PA Alliance – joint initiative between health department of NY and industry	Model guidelines for use in after school settings as a way to improve PA and obesity rates in children in New York.	Elementary and high school students

*U.S*. United States, *PA* Physical Activity, *NAA* National Afterschool Association, *HEPA* Healthy Eating and Physical Activity, *OSNAP* Out-of-School Nutrition and Physical Activity Initiative, *NY* New York, *YMCA* Young Men’s Christian Association, *OST* Out of School Time, *n/a* Not available

**Table 4:** Summary of PA and screen time recommendations during after school care sessions

Guideline Title	PA Recommendations		Screen time Recommendations		Guideline in use?
	**Duration**	**Intensity**	**Duration**	**Content**	
The National Afterschool Alliance HEPA Standards 2.0	•Plan and provide PA: a)1 h program = 10 mins b)2 h program = 20mins c)3 h program = 30mins d)4 h program = 40 mins e)5 h program = 60 mins	•MVPA for 50% of PA time (e.g. 5 mins of 10 min spent in MVPA)	•Daily total screen time is limited to: a)1- to 2-h. programs—40 mins b)3 h. or more—60 mins	•Ensure that digital devices are used for homework, research, or digital learning that is active rather than passive •No television or movies are allowed	YES
Before-And-After School Programs (Kindergarten to Grade 6) - Policies and Guidelines for School Boards	30 min of daily active play in daily programming to align with government initiatives	n/a	n/a	n/a	YES
Minnesota Afterschool and OST - Best Practice Guidelines	•30 min daily^a^ •20 min × 3/week ^a^	•MVPA^a^ •Vigorous^a^	•Limit recreational computer time to less than one hour a day^a^	•Eliminate the use of commercial broadcast TV/movies ^a^	YES
	•Schedule at least 30 min of for every 3-h block, and half of that time spent outside daily^b^	•MVPA^b^	•Limit television and recreational screen time to no more than 2.5 h of a 5-day week i.e. 30 min per day^b^		
Ohio Kids on the Move: Physical Activity Guidelines for Afterschool Programs	•For an academic /arts /science /community based after school program: 20% of the total session time dedicated to PA •For a physical activity based after school program: 80% of session time dedicated to PA	•MVPA	Screen time is limited to 10% of total program and no more than 20 min continuously.	n/a	YES
National Afterschool Association HEPA Standards	•For an academic /arts /science /community based after school program: 20% of the total session time dedicated to PA •For a PA based after school program: 80% of session time dedicated to PA	n/a	•Screen time is limited to 10% of total program time and no more than 20 min continuously	n/a	NO
OSNAP	•Include 30 min daily	•MPA, that is fun (include outdoor activity if possible)	•Limit recreational computer time to less than one hour a day	•Eliminate the use of commercial broadcast TV/movies	YES
	•20 min, 3 x week	VPA			
California After School Physical Activity Guidelines	•A minimum of 30–60 min •For students not engaging in PA elsewhere, aim to provide the full 60 min of recommended MVPA •Arrange the afterschool schedule to ensure that students do not sit for more than 60 min at a time	MVPA	•Limit recreational screen time to 30 min •Limit total screen time to 60 min per after school session	n/a	YES
Move More North Carolina: Recommended standards for After School Physical Activity, North Carolina	•20% of the total session time should be dedicated to PA in traditional/enrichment/academic focus programs •80% of the total session time should be dedicated to PA in sport/recreation focus programs	MVPA	n/a	n/a	YES
Healthy Kids, Healthy New York - After-School initiative toolkit	•Schedule at least 30 min of for every 3-h block, and half of that time spent outside daily	•MVPA	•Limit television and recreational screen time to no more than 2.5 h of a 5-day week i.e. 30 min per day	n/a	YES
	•Provide an activity break for no more than 60 min of continuous sedentary activity				

*PA* Physical Activity, *AS* After School, *MVPA* Moderate to vigorous physical activity, n/a Not available

*U.S*. United States, *NAA* National Afterschool Association, *HEPA* Healthy Eating and Physical Activity, *OSNAP* Out-of-School Nutrition and Physical Activity Initiative, *NY* New York, *YMCA* Young Men’s Christian Association, *OST* Out of School Time, *n/a* Not available ^a^From the OSNAP guidelines, ^b^ From the NY guidelines

The included guideline documents were developed by either a department of education (*n* = 2), a collaboration between a department of health and private sector/after school network (n = 2), a collaboration between a University centre with government funding; private sector and/or after school sector and/or non for profit e.g. YMCA (*n* = 4); or a collaboration between a department of health, a university, private sector, afterschool network and families (*n* = 1).

## Synthesis of results

### Methods for creation of guidelines

Eight of the nine included guideline documents used a similar method to create their guidelines. This consisted of a panel of experts including research personnel, industry personnel (e.g. OSHC directors, facilitators) and government authorities. It is clear from five of the guidelines that key stakeholders were also included in the development process in (e.g. The Move More North Carolina [37] guidelines additionally collaborated with parents, administrators, funders and community partners). Funding came from a variety of sources, with three of the guideline documents funded by government departments and five funded through a combination of industry and universities. The Minnesota guidelines [32] did not “create” their own guidelines as such, so did not require funding. Rather, they summarised and collated the current guidelines from the Out of School Nutrition and Physical Activity (OSNAP) [35], Healthy Eating and Physical Activity (HEPA) [34], New York State Healthy Eating and Physical Activity Alliance (NY) [38] and Move More North Carolina guidelines (MMNC) [37]. The methods used to create the Ontario Ministry of Education guidelines [31] are unclear as no details of methods or contact details / corresponding author were provided.

### Summary of evidence

#### PA recommendations in the guidelines

##### Before school care

Before school care session guidelines were limited to two guideline documents. The HEPA 2 [30] recommend OSHC “dedicate at least 20% or at least 30 minutes of morning or afterschool program time to physical activity” p.5. Whilst the Ontario guidelines [31] do not explicitly state how much should be allocated for morning or afternoon programming, but clearly state in the title the guidelines are for “Before-and-After School Programs …” and that “a minimum of 30 minutes of active play in daily programming to align with the Government’s commitment …” p.10.

##### After school care

For a typical three hour afternoon care session, the recommendations across nine guidelines ranged between 30 and 60 min of PA. Six of the nine guidelines had a simple fixed recommendation for example, the OSNAP guidelines [35] recommended inclusion of “30 minutes of moderate, fun, PA for every child, everyday”. Three of the guidelines recommended that PA time vary according to the length of a session (*n* = 3), for example the MMNC guidelines [37] recommended that 20% of the total session time should be allocated to PA and provides an example of how to calculate this and suggested activities to ensure children are engaging in MVPA rather than light PA. The HEPA guidelines update [30] goes beyond this, providing varying recommendations per session length, and separate recommendations for light PA and MVPA. The HEPA guidelines [30] recommend that for the time allocated to PA, 50% of PA time should be spent in MVPA (i.e. for a 1 h program, 10 min is for PA, of which 5 min should be MVPA).

##### Screen time recommendations in the guidelines

Seven of the included guidelines also provided screen time recommendations, of which six were focused on the after school period [32–36, 38], and one provided recommendations for both the before and after school periods [30]. Four of the guidelines had a simple recommendation of the maximum duration of screen time. For example, the Minnesota guidelines [32] recommended that recreational computer time is limited to less than one hour a day. Whilst the HEPA 2 [30], HEPA 1 [34] and Ohio [33] guidelines provided recommendations that varied depending on the session length of the after-school session (*n* = 3): for example, the Ohio Afterschool Network recommended that screen time is limited to 10% of total program time [33]. Some of the recommendations provided advice regarding the screen content e.g. discouraging the use of commercial broadcast TV/movies [35] as summarised in Table 4. Similarly, the 2018 updated HEPA 2 guidelines provided a varying time frame of screen time use dependent on session length, in addition to recommendations on the use of digital devices (i.e. for homework only) and specifically that no television or movies should be allowed [30].

##### Age group

The age range for which these guidelines were written for was only clearly described in four of the guideline documents [31, 35, 37, 38]. The Ontario guidelines [31] were written specifically for children aged Kindergarten (approximately age 5) to Year 6 (approximately age 12). The OSNAP guidelines [35] were written for children aged 5–12 years (elementary school), whilst the MMNC [37] and NY [38] guidelines state they were for children and youth. The remaining 5 guideline documents did not specify the age limits (see Table 3).

## Discussion

This scoping review found nine documents that provided guidelines for PA in the OSHC setting. All targeted the after school care session, whilst two of these nine also targeted before school care [30, 31]. All provided a target for PA ranging from 30 to 60 min. Seven also provided a recreational screen time recommendation of no more than 60 min. In addition, all were published in grey literature and all were developed by expert panels comprising of a variety of stakeholders.

The amount and type of PA recommended from each of the guidelines varies. Three of the guideline documents recommended the amount of PA in terms of a percentage of the session [30, 34, 37]; whilst four specified an amount of time (i.e. 30 min [31–33, 35] and one recommended a range [36]. In addition to the time variation, there was variation in intensity guidelines, with some referring to “active play” without specify intensity [31], most referring to MVPA [30, 32–36] and two guidelines providing specific targets for vigorous PA in addition to MVPA [32, 35]. This variation in time and intensity means that implementing such guidelines across afterschool programs to increase PA could be difficult, due to the different aims of each guideline. Whilst the recommendation of 30–60 min of PA is congruent with 24-h daily guidelines, the lack of consistency may make implementation difficult. This is in contrast to whole day guidelines which are internationally consistent [39].

Conversely, the duration of screen time recommended was more consistent. Of the seven guideline documents that had screen time recommendations, four clearly stated no more than 60 min of recreational screen time should be allowed [30, 32, 33, 35]. The only variation from this came from the Californian guidelines [36] which had a much shorter limit on screen time, of no more than 30 min. The superceded HEPA 1 [34] standards provided screen time as a percentage of time, rather than a fixed amount of time. Given this was their older recommendation, it appears that this ambiguity was recognised hence the more refined recommendations in their recent guideline document [30]. These screen time recommendations also align well with 24-h daily movement guidelines (which recommend no more than 2 h a day of recreational screen time).

All but one of the guidelines were developed in consultation with an expert panel, typically comprising of representatives from industry (e.g., care staff), government (e.g., Education Department, Health Department), non-government bodies (e.g., YMCA) and academics. Only one guideline clearly stated that parents were involved in the guideline development process [37]. The guideline developers typically reported that they consulted the scientific literature, however none of the guidelines appeared to follow “gold-standard” methodologies for guideline development, such as the Grading for Recommendations Assessment, Development and Evaluation (GRADE) approach [40] or Guidelines-International-Network (GIN) [41]. In recent years, these methodologies and tools have been more widely adopted in clinical and health service contexts to improve the quality of the guidelines and their implementation [42]; however they are yet to be widely adopted in public health/education. This may explain why these methodologies were not used for any of the OSHC guidelines. Presumably in time, other jurisdictions will produce guidelines for PA and screen time in OSHC. Also, future guideline development could incorporate guideline development methodologies such as GRADE [40], G-I-N [41] or AGREE [43]. These methodologies provide a systematic approach to using latest evidence and consulting widely with stakeholders with a view to maximising implementation, the fundamental goal of guidelines [44].

Whilst these guidelines serve as a starting point for future works, to help improve practice in this setting, there are some limits to the guidelines themselves. Unlike the 24-h movement guidelines for children aged 5–17 years, which have clear and consistent recommendations for the amount of MVPA, Vigorous PA, light PA, sedentary, recreational screen and sleep, there is inconsistency in the duration and intensity of PA recommendations in the OSHC guidelines identified in this scoping review. Whilst it would be inappropriate for an OSHC guideline to target sleep, it would be valuable to have more clear and consistent messaging on the duration and intensity of PA that is being targeted. Likewise, the OSHC guidelines found for this review did not have a consistent age range. The Ontario guidelines are for children from Kindergarten (approximately age 5) to Year 6 (approximately age 12) and it is clearly stated in the title [31]. Three other guidelines [30, 34–36] state they are for children and adolescents or youth (up to age 17), however one of the guidelines has accompanying published papers which implemented the guidelines on children aged 5–12 or elementary school aged children [45]. The remaining four guideline documents do not specify an age range. This ambiguity, may make implementation or adolopment [46] of these guidelines difficult. In addition, there is no mention given in any of these guidelines regarding school holidays PA and screen time recommendations; when children are in OSHC for a full day, rather than a short period before or after school.

In addition to this, a small number of studies have examined implementation of guidelines. Gortmaker et al. [47] examined the OSNAP guidelines (the food and fun component) and found that controlling for baseline covariates, children in intervention sites showed greater increases in average PA level than in control sites. Other research conducted more recently by Beets et al. [48] and Weaver et al. [49] investigated the effectiveness of the Californian guidelines and HEPA guidelines. Results suggested that guideline implementation led to an increase in MVPA in boys but not girls. This raises the issue that perhaps such guidelines need to address how to encourage PA for girls, in addition to the entire cohort of children attending OSHC.

### Strengths and limitations of the review

Strengths of this review include that it was prospectively registered, and a search strategy that covered both scientific and grey literature was implemented. In addition, it is a novel scoping review, with the only other review of OSHC guidelines having come from Beets et al. [19]. By contrast that earlier review only assessed local policies and guidelines and did not attempt to gauge an international perspective. There are some limits to the results of this review too. The search strategy used English terminology, and so there may be non-English guidelines that were not identified by our search strategy (though we are not aware of any). Likewise, all guidelines originated from North American jurisdictions, thus their generalizability to other world regions and cultures is unclear.

## Conclusion

To date, relatively few guidelines addressing PA and screen time patterns in OSHC settings have been published. Existing guidelines have originated from North America. These guidelines collectively recommend 30–60 min of PA and no more than 60 min of recreational screen time during after school care sessions, and 30 min of PA is recommended for the before school session. Future efforts should consider PA and screen time both during the before school and after school care periods and may benefit from following rigorous guideline development processes. In addition, efforts to implement and evaluate the effectiveness of implementation strategies are warranted.

## Supplementary information

**Additional file 1.**

## Data Availability

The datasets used and/or analysed during the current study are available from the corresponding author on reasonable request.

## Abbreviations

PA: Physical activity
OSHC: Outside school hours care
MVPA: Moderate- to- vigorous physical activity
PRISMA-ScR: Preferred Reporting Items for Systematic Reviews and Meta-analysis
